# Twelve year trajectories of physical activity and health costs in mid-age Australian women

**DOI:** 10.1186/s12966-020-01006-6

**Published:** 2020-08-10

**Authors:** Grace A. O. Gomes, Wendy J. Brown, Jamile S. Codogno, Gregore I. Mielke

**Affiliations:** 1 Department of Gerontology, Federal University of São Carlos, Rod. Washington Luiz, s/n, São Carlos, SP 13565-905 Brazil; 2 School of Human Movement and Nutrition Sciences, The University of Queensland, St Lucia QLD, Brisbane, Queensland 4072 Australia; 3 Department of Physical Education, Presidente Prudente, São Paulo State University, R. Roberto Símonsen, 305 - Centro Educacional, Pres. Prudente, SP 19060-900 Brazil

**Keywords:** Exercise, Behavior, Health care costs, Health expenditures, Middle aged, Older adults

## Abstract

**Background:**

Few studies have examined relationships between physical activity (PA) during mid-age and health costs in women. The aim of this study was to investigate associations between PA levels and trajectories over 12 years with medical and pharmaceutical costs in mid-age Australian women.

**Methods:**

Data from 6953 participants in the Australian Longitudinal Study on Women’s Health (born in 1946–1951) were analysed in 2019. PA was self-reported in 2001 (50-55y), 2007 (56-61y) and 2013 (62-67y). PA data were linked with 2013–2015 data from the Medicare (MBS) and Pharmaceutical (PBS) Benefits Schemes. Quantile regression was used to examine associations between PA patterns [always active, increasers, decreasers, fluctuaters or always inactive (reference)] with these medical and pharmaceutical costs.

**Results:**

Among women who were consistently inactive (< 500 MET.minutes/week) in 2001, 2007 and 2013, median MBS and PBS costs (2013 to 2015) were AUD4261 and AUD1850, respectively. Those costs were AUD1728 (95%CI: 443–3013) and AUD578 (95%CI: 426–729) lower among women who were consistently active in 2001, 2007 and 2013 than among those who were always inactive. PBS costs were also lower in women who were active at only one survey (AUD205; 95%CI: 49–360), and in those whose PA increased between 2001 and 2013 (AUD388; 95%CI: 232–545).

**Conclusion:**

Maintaining ‘active’ PA status was associated with 40% lower MBS and 30% lower PBS costs over three years in Australian women. Helping women to remain active in mid-life could result in considerable savings for both women and the Australian government.

## Background

The health benefits of physical activity (PA) are well known; they include prevention and control of weight gain, non-communicable diseases and mental health problems [1, 2]. Despite these benefits, it is estimated that globally only one in four adults achieve the current PA guidelines [3, 4], and women are more inactive than men in most countries [3–5].

Although maintaining PA at recommended levels over time is essential for women’s health, PA levels change continually across the lifespan. Data from the Australian Longitudinal Study on Women’s Health show that when women transition from ages 50–55 to 53–58, only one third maintain recommended levels of PA over 3 years, whereas one third remain inactive, and one third change from active to inactive or vice-versa [6]. Moreover, there are marked declines in PA among young married women with children, mirroring the constraints imposed on available time for PA, due to paid and unpaid work commitments. Following retirement from the paid workforce, and when children leave home, some women increase their PA, but in older age PA levels tend to decrease as health problems increase [3, 6–9]. Overall, there is a trend of declining PA with increasing age; both are associated with worsening health outcomes [10].

Recently published research has shown that mid-age and older adults can gain substantial health benefits by becoming more active, regardless of past PA levels and established risk factors [11–13]. Other studies have shown positive impacts of becoming more active on intermediate health outcomes at older age, such as physical function [14], joint symptoms [15] and cognitive function [16]. These positive effects may influence utilization of health services in later years, and consequently, the costs. Although several cross-sectional studies support the evidence of associations between PA and use and costs of health services [17–21], only one US study [22] and two previous analyses of ALSWH data [23, 24] have examined these relationships prospectively.

Longitudinal associations between PA levels and trajectories with health costs among mid-age women are still largely unexplored. Therefore, the aim of this study was to investigate PA levels and trajectories over 12 years (when the women were in their early 50s to mid-60s) and their associations with medical and pharmaceutical costs 3 years later among Australian women. We hypothesized that being more physically active during mid-age would be associated with lower medical and pharmaceutical costs in later life.

## Methods

### Design and participants

This study used data from the mid-age cohort (born in 1946 to 1951) of the Australian Longitudinal Study on Women’s Health (ALSWH), an ongoing study of Australian women. Baseline surveys were mailed in 1996 when women were 45–50 years old (*N* = 13,714), with follow up surveys from 1998 to 2016 at 3 year intervals. At baseline, the sample was largely representative of Australian women in this age group, but with a somewhat higher representation of partnered women and women with post-high school education [25]. Ethics approval for the study was gained from the relevant ethics committees at the University of Newcastle (No. H-2010_0031) and the University of Queensland (No. 2010000411). Participants gave their informed consent to participate. Further details of the recruitment methods and response rates and data collection have been described elsewhere [25]. For this study, we analysed data from women with PA data in 2001 (50-55y), 2007 (56-61y) and 2013 (62-67y), and who consented to linkage with healthcare administrative data (*N* = 6953).

### Physical activity

Physical activity (explanatory variable) was assessed in 2001, 2007 and 2013 using a modified version of the Active Australia questionnaire, which has acceptable reliability and validity for use in cohort studies [26]. Participants were asked about frequency and duration of walking briskly (for recreation or exercise or to get to or from places), moderate-intensity leisure- activities (like social tennis, moderate exercise classes, recreational swimming, dancing), and vigorous-intensity leisure-time activities (that make you breathe harder or puff and pant) in the last week. They were asked to only report PA that lasted 10 min or more. Minutes per week spent in each activity were multiplied by a metabolic equivalent (MET) score: 3.33 for walking and moderate intensity leisure activities and 6.66 for vigorous leisure-time activity. The amount of PA was calculated as the sum of MET-minutes/week from each of the domains and categorised as: none (0 − < 33.3; reference category); low (33.3 − < 500); moderate (500 − < 1000); or high (≥1000) [27].

### Costs of health services

Health costs data from the Australian Medicare Benefits Scheme (MBS) and the Pharmaceutical Benefits Scheme (PBS) were the outcome measures for these analyses. Total health costs, which include the government benefits paid and the out-of-pocket costs paid by the patient for each service (MBS) or prescription (PBS), were calculated for each participant as the sum of total costs from 2013 (62-67y) to 2015 (64-69y). MBS is the Australian government’s system for subsidising general practitioner and some out-of-hospital specialist, pathology, radiology, dental and allied health services, and limited additional primary healthcare services, for all Australian citizens and permanent residents (MBS, 2019) [28]. PBS is the system that subsidises the cost of approved prescribed medications.

### Covariables

In 2013, participants provided information on age, area of residence, education, marital status, smoking, alcohol, and perceived health. Alcohol status was based on questions about frequency and quantity of alcohol intake [29] and all variables were categorised as shown in Table 1. The women were also asked whether they had a health care card (which allows disadvantaged patients lower out-of-pocket costs). Body Mass Index (BMI) was categorized according to the World Health Organization classification: underweight (BMI < 18.5 kg/m^2^); normal weight (BMI 18.5–24.9 kg/m^2^), overweight (BMI 25.0–29.9 kg/m^2^) or obese (BMI ≥ 30 kg/m^2^). In each survey, women were asked “In the past three years, have you been diagnosed or treated for...” followed by a list of conditions which are common in mid-age women (including arthritis, diabetes, heart disease, asthma, breast and colon cancer, depression, anxiety etc). Copies of the surveys are available at www.alswh.org/surveys.

**Table 1:** Sociodemographic and health characteristics of the analytical sample in 2013, and health costs in 2013–2015, according to **s**ociodemographic and health characteristics (*N* = 6953)

**Characteristics**			**Health Costs 2013–2015 (AUD)**^**a**^	
	**N**	**%**	**MBS**^**b**^ **Median (25th -75th)**	**PBS**^**c**^ **Median (25th -75th)**
**Area of residence**				
Urban	2694	39.3	4275 (2089–7816)	1156 (303–2647)
Rural	3927	57.3	3645 (1849–6624)	1330 (360–2991)
Remote	233	3.4	2169 (208–5244)	883 (0–2358)
**Education**				
No f ormal education	743	11.5	3833 (1942–6775)	1744 (585–3498)
School certificate	3052	47.2	3816 (1930–7016)	1391 (400–3067)
Higher school certificate	2666	41.2	3582 (1747–6791)	905 (185–2321)
**Marital status**				
Married/de facto	5245	75.8	3802 (1877–7028)	1217 (309–2730)
Separated/divorced/widowed	1497	21.6	3762 (1778–6871)	1358 (328–3225)
Never married	177	2.5	4189 (2151–8174)	1313 (294–3089)
**Health care cards**				
Yes	3302	47.6	4076 (2029–7409)	1788 (294–3089)
No	3626	52.3	3579 (1714–6665)	828 (172–2093)
**Body mass index**				
Underweight / Normal	2438	36.2	3463 (1655–6453)	711 (135–1906)
Overweight	2292	34.1	3692 (1783–6757)	1254 (321–2616)
Obese	1992	29.6	4437 (2235–7975)	2096 (871–4090)
**Smoking status**				
Never-smoked	4374	63.1	3801 (1871–7015)	1155 (284–2621)
Ex-smoker	2125	30.6	3891 (1946–7132)	1353 (370–3104)
Smoker	431	6.2	3315 (1361–6276)	1649 (429–4041)
**Alcohol**				
Low risk drinker	3849	55.7	3626 (1794–6834)	1011 (242–2329**)**
Non-drinker	1084	15.7	4064 (1951–7562)	1781 (457–3979)
Rarely drinks	1560	22.6	4080 (1968–7562)	1532 (429–3390)
Risky/high risk drinker	411	5.9	3544 (1601–6428)	1346 (461–2879)
**Self-reported health**				
Excellent/very good	3342	48.1	2946 (1440–5519	668 (125–1698)
Good	2707	39.0	4273 (2236–7537)	1690 (623–3381)
Fair/poor	891	12.8	6530 (3540–10,988)	3812 (1658–7125)
**Number of chronic health conditions**				
0	1301	19.6	2076 (967–4011)	214 (33–813)
1–2	3671	55.3	3590 (1868–6430)	1137 (352–2272)
≥ 3	1663	25.0	5931 (3331–9964)	3015 (1530–5582)

^a^AUD = Total health costs over three years in Australian Dollars (2013–2015);

^b^ MBS = Medical Benefits Scheme;

^c^ PBS = Pharmaceutical Benefits Scheme

### Data analysis

Sociodemographic and behavioural characteristics of the analytical sample in 2013 were summarized using descriptive statistics (eg proportions, medians and Interquartile ranges -IQRs). Median and IQRs were calculated for MBS and PBS health costs during 2013–2015 according to sociodemographic characteristics in 2013.

For the trajectory analyses women were categorised as either ‘inactive’ (none or low) or ‘active’ (moderate or high) at each survey. The proportion of active women at each survey was calculated and changes in activity status (active/inactive) between 2001 and 2007 and between 2007 and 2013 were computed and illustrated using a lasagne plot [30]. To elucidate the associations between physical activity and health costs, the analyses were conducted in four steps. First, median and interquartile ranges for total MBS and PBS costs in 2013–2015 were calculated for each physical activity category in 2001, 2007 and 2013. Second, costs were calculated for five groups according to PA trajectories: a) always inactive (inactive in all three surveys); b) always active (active in all three surveys); c) increasers (changed from inactive in 2001 to active in 2013); d) decreasers (changed from active in 2001 to inactive in 2013); e) ‘fluctuaters’ (classified in the same PA category in 2001 and 2013, but in the opposite category in 2007). Third, a cumulative PA score was created by summing the number of times women were categorized as active (0, 1, 2 or 3) and costs were calculated for women in each of these categories. Fourth, crude and adjusted quantile regression models were used to estimate the differences in median costs for each of the PA variables described in steps one to three. Analyses were adjusted for confounders that have been shown in previous ALSWH studies to be associated with both PA and health costs [15, 24, 31]. These included age, education, marital status, area of residence, having a health care card, smoking, alcohol and BMI. Associations between physical activity trajectories and sociodemographic and health characteristics are shown in Supplementary Table 1. Number of chronic conditions was not included in the regression models because many of the chronic conditions could be mediators of the association between PA and medical and pharmaceutical costs. All statistical analyses were performed using Stata 16.1 in 2019.

## Results

Of the 13,714 women who were age 45–50 years when the cohort was established in 1996, 12,707 (92.7%) consented to data linkage. Of these, 4915 women did not complete at least one of the three surveys (2001, 2007, and 2013) and 839 were excluded because they had missing information for PA in one or more surveys. The analytical sample included 6953 women (Supplementary Figure 1).

The women’s sociodemographic and health characteristics are presented in Table 1. Their average age in 2013 was 64.5 (range 62–67 years), nearly two thirds were overweight/obese, 6% were current smokers and one quarter had three or more chronic health conditions. Those whose data were included in the analyses were more likely to have post-school education, excellent/very good perceived health and to be active, and less likely to have a health care card, than those who were not included in the analyses due to loss-to-follow up or missing data (*n* = 6761). (Supplementary Table 2).

**Fig. 1:**
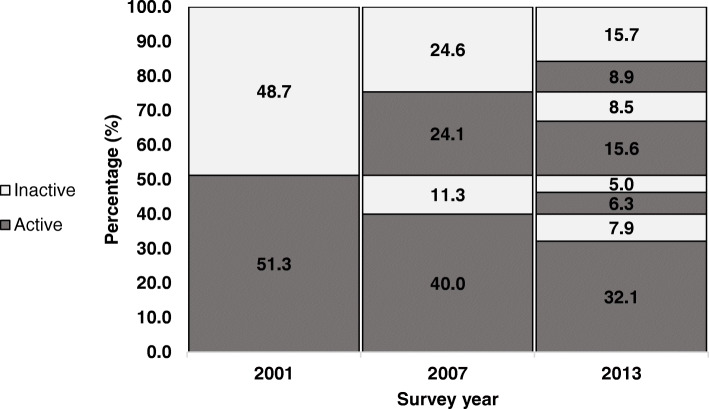
Lasagne plot to illustrate changes in physical activity category (2001, 2007, 2013) in mid-age women (*N* = 6953). The data show, for example, that 11.3% of women were active in 2001 but inactive in 2007, and 7.9% were active in the first two surveys and inactive in 2013, etc.

The distributions of PA at each survey are presented in Fig. 1, and PA trajectories are shown in the middle panel of Table 2. Approximately half the women were active in 2001. This proportion increased by 13 percentage points from 2001 to 2007 (64.1%), then remained fairly stable from 2007 to 2013 (62.9%). More than half the women changed their PA status over the three time points, about one third were active, and 15.7% were inactive at all three surveys. One in four women increased their PA from 2001 to 2013, whereas 13% decreased their PA in this period; 22.4% were active in only one of the three surveys.

**Table 2:** Estimates of MBS and PBS costs in 2013–2015 according to physical activity indicators from 2001 to 2013 in mid-age Australian women (*N* = 6953)

				**Health Costs (AUD) 2013-2015**^**a**^	
**Physical activity**				**MBS** ^**b**^	**PBS** ^**c**^
	**N**	**%**		**Median (25th -75th)**	**Median (25th -75th)**
**2001 (50–55 years)**					
None	1047	15.1		4355 (2204; 7774)	1851 (678; 4000)
Low	2341	33.6		3726 (1895; 6936)	1321 (323; 2955)
Moderate	1575	22.7		3660 (1787; 6675)	1034 (285; 2461)
High	1990	28.6		3737 (1734; 6934)	1026 (212; 2500)
**2007 (56–61 years)**					
None	945	13.6		4355 (2071; 7816)	1991 (717; 4351)
Low	1547	22.3		3697 (1862; 6948)	1306 (325; 2985)
Moderate	1670	24.0		3741 (1976; 6688)	1264 (328; 2697)
High	2791	40.1		3689 (1722; 6880)	999 (221; 2424)
**2013 (62–67 years)**					
None	1135	16.3		4989 (2534; 9217)	2093 (848; 4729)
Low	1448	20.8		3895 (2030; 7134)	1493 (443; 3110)
Moderate	1468	21.2		3562 (1830; 6632)	1055 (240; 2556)
High	2902	41.7		3444 (1646; 6441)	947 (205; 2262)
**Trajectories of PA**					
Always inactive	1092	15.7		4261 (1991; 7623)	1850 (647; 4052)
Always active	2233	32.1		3448 (1677; 6350)	818 (177; 2066)
Increasers	1702	24.4		3508 (1765; 6661)	1183 (300; 2646)
Decreasers	897	13.0		4542 (2226; 8219)	1692 (538; 3456)
Fluctuaters	1029	14.8		4170 (2089; 7328)	1460 (367; 3224)
**Number of surveys as active**					
0	1092	15.8		4261 (1991; 7623)	1850 (647; 7458)
1	1559	22.4		4098 (2225; 7644)	1569 (468; 3358)
2	2069	29.7		3823 (1761; 6937)	1216 (315; 2768)
3	2233	32.1		3448 (1677; 6350)	818 (177; 2066)

^a^ AUD: Health costs over three years in Australian Dollars (2013–2015)

^b^ MBS: Medical Benefits Scheme

^c^ PBS: Pharmaceutical Benefits Scheme

The median MBS and PBS costs from 2013 to 2015 were AUD3,788 (IQR: 1859-7015) and AUD1245 (IQR: 315–2843) respectively. Overall, these costs were consistently higher among women in the lowest PA category, at every survey (see top panel of Table 2). The median costs were higher among women who were always inactive than in those who were always active. Costs were markedly lower in the increasers than in the decreasers or fluctuaters (see centre panel of Table 2). Both MBS and PBS costs declined with increasing numbers of surveys in which women were categorized as active (see bottom panel of Table 2).

Differences in median 3 year costs between categories of PA in each survey (2001, 2007 and 2013), PA trajectories, and the number of surveys women were active, are presented in Table 3. In adjusted models, median MBS costs were similar across the PA categories in 2001 and 2007, but in 2013 costs decreased with increasing PA. The median cost was AUD3,522 (95%CI: 2212–4831) lower in women with high levels of PA in 2013 than among women who were inactive. In women who were always active, costs were AUD1,728 lower than in those who were always inactive (across three surveys in 12 years; see middle panel of Table 3).

**Table 3:** Differences in median MBS and PBS costs (2013–2017) for categories of PA variables (levels and trajectories) from 2001 to 2013 in mid-age Australian women. (*N* = 6953)

	**Health Costs 2013–2015 (AUD) **^**a**^			
**PA level**	**MBS** ^**b**^		**PBS** ^**c**^	
	**β**_**crude**_ **(95%CI)**	**β**_**adjusted**_ **(95%CI)** ^**d**^	**β**_**crude**_ **(95%CI)**	**β**_**adjusted**_ **(95%CI)** ^**d**^
**2001 (50–55 years)**				
None	0	0	0	0
Low	− 628 (− 969; −287)	− 507 (− 1840; 825)	− 529 (−690; − 368)	− 363 (− 515; − 211)
Moderate	−695 (− 1060; −329)	− 1106 (− 2537; 323)	−817 (−989; −644)	− 531 (−694; −367)
High	− 615 (− 965; −265)	− 719 (− 2096; 658)	−823 (−988; − 657)	−508 (−665; −351)
**2007 (56–61 years)**				
None	0	0	0	0
Low	−658 (− 1049; − 267)	− 138 (− 1638; 1362)	− 684 (− 875; −493)	− 377 (− 541; −212)
Moderate	− 612 (− 997; −226)	−243 (− 1730; 1243)	− 727 (−915; −538)	− 372 (−535; −209)
High	−666 (− 1022; −310)	−632 (−2014; 749)	−992 (− 1166; − 818)	−544 (− 696; −391)
**2013 (62–67 years)**				
None	0	0	0	0
Low	− 1090 (− 1448; −733)	− 2565 (− 4023; − 1107)	− 598 (− 755; − 442)	− 320 (− 485; −155)
Moderate	− 1426 (− 1782; 1069)	− 3437 (− 4898; −1976)	− 1034 (− 1210; − 858)	− 605 (− 771; − 439)
High	− 1544 (− 1860; − 1228)	− 3522 (−4831; −2212)	− 1145 (− 1301; − 989)	− 669 (− 818; −519)
**PA trajectories**				
Always inactive	0	0	0	0
Always active	−813 (− 1148; −477)	−1728 (−3013; −443)	− 1032 (− 1195; − 870)	−578 (−729; −426)
Increasers	− 750 (− 1102; −397)	− 1117 (− 2448; 212)	− 666 (− 837; − 495)	−388 (−545; −232)
Decreasers	281 (− 128; 690)	1040 (− 499; 2579)	− 158 (− 357; 39)	−130 (− 312; 50)
Fluctuaters	−91 (486; 303)	− 434 (− 1919; 1049)	− 391 (− 582; − 199)	−260 (− 434; − 86)
**Number of surveys active**				
0	0	0	0	0
1	−163 (− 540; 214)	245 (− 1162; 1653)	− 282 (− 449; − 114)	−205 (− 360; − 49)
2	−438 (− 796; − 80)	− 811 (− 2152; 529)	− 634 (− 793; − 475)	− 382 (− 530; − 234)
3	−813 (− 1166; − 459)	− 1771 (− 3112; − 430)	− 1032 (− 1189; − 875)	−585 (− 734; − 437)

^a^ AUD: Total health costs over three years in Australian Dollars (2013–2015)

^b^ MBS: Medical Benefits Scheme

^c^ PBS: Pharmaceutical Benefits Scheme

^d^ Adjusted for: Age, area, marital status, education, health card, smoking alcohol and BMI

At each survey, women who reported at least low levels of physical activity had lower PBS costs from 2013 to 2015 than women who were inactive (Table 3), and costs decreased further with increasing PA category. In the trajectory analyses, PBS costs were AUD578, AUD388, and AUD260 lower in women who were always active, those who increased PA levels and ‘fluctuaters’ over 12 years, respectively, than for women who were always inactive. There was a dose-response relationship between number of surveys that women were active and PBS savings. Women who were active in at least one survey had AUD205 lower PBS costs from 2013 to 2015 than women who were always inactive (see bottom panel of Table 3).

## Discussion

This study provides novel insights into levels and trajectories of physical activity during mid-age and their associations with health costs in mid-age Australian women. Our findings show an overall increase in PA levels from 2001 (50–55 years) to 2007 (56–61), which was maintained in 2013 (62–67). Consistent with previous studies, fewer than half the participants remained in the same PA category over 12 years, but those who were consistently active in this period had lower MBS and PBS costs than those who were inactive.

Our findings are consistent with reports from several studies conducted in other countries, which have shown inverse relationships between PA and health costs [19, 32–37]. However, no previous studies have examined the costs associated with trajectories and changes in PA. This is important, because physical activity changes throughout the lifespan, often in parallel with important life events (e.g. having a baby or widowhood) and changing social circumstances (e.g. paid work, retirement) [6, 38–41]. In contrast with our finding that only one third of these mid-age women were consistently active, Smith et al. [9] reported that almost half (49%) of those over 65 years in a UK sample were persistently active over 10 years. However, differences in the number of time points at which PA is measured, as well as in the instruments used, and the age and gender of participants, mean that comparisons should be made with caution.

Because of substantial increases in the use of healthcare resources in recent decades, in particular among older populations, governments worldwide are attempting to determine strategies for controlling utilization of healthcare and consequent health costs [42, 43]. Focussing on PA in mid-age may be particularly important, because this is a life-stage when risk of disability and complications from non-communicable diseases begin to increase. Our data suggest that strategies to increase physical activity in mid-age could save expenditure for governments, as well as for women themselves.

As the overall cost of inactivity internationally was estimated to be INT$ 53.8 billion worldwide per annum in 2013 [34], one approach to reducing health costs may be to increase population levels of PA. Our findings show that maintaining high active status was associated with 30–40% lower costs after 12 years, in a period when health costs in Australia increased by 2.7 and 3.6% for primary health care and hospitals, respectively [44, 45]. We estimated that 32% of women were always active and there were cost savings of AUD1728 for MBS and AUD578 for PBS every 3 years for these women. At the population level (with 2,914,000 women in the 50–69 years age group in Australia), and assuming there is a causal relationship in our analyses, we estimate that this would represent a saving of AUD2306 for 932,480 women, which equates to AUD2.15bn over 3 years.

The strengths of this study include the use of a large population-based sample of mid-age women followed over 12 years, and linkage with government data on the costs of both medical and pharmaceutical services. The large analytical sample allowed us to estimate health costs for a range of trajectories of PA, after adjustment for established risk behaviors and sociodemographic characteristics that influence health costs. Further, the use of time lagged analyses showed consistent associations between low levels of PA at different time points and higher health costs some years later. Finally, because the PA trajectories (increasers, decreasers and fluctuaters) included different numbers of women who were active at each time, we also analysed the associations between PA patterns and health costs by the number of surveys the women were active; the results were only marginally changed.

As with all observational cohort studies, there are some limitations. Firstly, women whose data were included in the analyses were more educated and healthier than women in the original cohort; this may mean that our overall health cost estimates are underestimated. Secondly, self-reported PA is subject to recall and social desirability bias, which can overestimate the prevalence of ‘active’ women. These biases are likely to be non-differential across the different levels of health costs, and would probably result in underestimation of the magnitude of the association between PA and health costs. Thirdly, while there was an element of ‘time lag’ in the analyses (ie PA data in 2001 and 2007 were linked with health cost data in 2013–2015), the associations between PA in 2013 and 2103–15 health costs may be subject to reverse causation, whereby women who were ill in 2013 had lower PA in that year. This would also have affected the trajectory analyses and the cost estimates. Finally, even though numerous confounding variables were included in the analyses, there may have been residual confounding due to unmeasured variables.

Given the expected increased risks in chronic conditions and consequently higher health expenditures with increasing age, our results point to the utility of increasing PA in mid-age to reduce health care costs in older age, for both individuals and the government. Continued efforts to facilitate the implementation of population-based PA promotion strategies are needed. Moreover, more longitudinal studies with robust, objective PA and health costs measures would enhance our understanding of the effects of PA on utilization and costs of healthcare services.

Our findings provide insights into the associations between changing patterns of PA and health costs among mid-aged women. Australian women who were consistently active at this life stage had markedly lower MBS and PBS costs. We conclude that, to achieve health expenditure savings for women in their 50s and 60s, it would be necessary for low active women to become more active and for active women to remain active, for as long as is possible across the lifespan. It is not too late for women in their sixties to become more active and save costs for themselves as well as the health system.

## Supplementary information

**Additional file 1: Supplementary Table 1.** Physical activity trajectories according to sociodemographic and health characteristics (*N*=6,953). **Supplementary Table 2.** Comparison of sociodemographic, health conditions and physical activity levels in the analytical sample and those lost to follow up. (All data are from the baseline survey in 1996, unless indicated)

**Additional file 2: Supplementary Figure 1.** Flow diagram showing the selection of participants for inclusion, Australia, 2001-2013

## Data Availability

The data that support the findings of this study are available from the Australian Longitudinal Study on Women’s Health, but restrictions apply to the availability of these data. De-identified survey data are available following an expression of interest (EOI) application through the ALSWH website (http://alswh.org.au/for-researchers) and approval by the ALSWH data access committee. The linked MBS and PBS data were used under license for the current study, and so are not publicly available. Linked analyses are also possible, subject to ALSWH EOI approval, but use of external linked datasets may only be conducted at the University of Queensland, the University of Newcastle or through the SURE facility at the Sax Institute https://www.saxinstitute.org.au/our-work/sure.

## Abbreviations

ALSWH: Australian longitudinal study on women’s health
BMI: Body mass index
PA: Physical activity
PBS: Pharmaceutical benefits scheme
MBS: Medical benefits scheme
