# Supplementary material for: Twelve year trajectories of physical activity and health costs in mid-age Australian women
Source: Int J Behav Nutr Phys Act. 2020 Aug 10;17:101. doi: 10.1186/s12966-020-01006-6 (PMC7418418; doi:10.1186/s12966-020-01006-6)
Supplement: Supplementary file 1 — Additional file 1: Supplementary Table 1. Physical activity trajectories according to sociodemographic and health characteristics (N=6,953). Supplementary Table 2. Comparison of sociodemographic, health conditions and physical activity levels in the analytical sample and those lost to follow up. (All data are from the baseline survey in 1996, unless indicated) [file 12966_2020_1006_MOESM1_ESM.docx]

**Supplementary Table 1:** Physical activity trajectories according to sociodemographic and health characteristics (N=6,953).

| **Characteristics** | N | Always inactive  % | Always active  % | Increasers  % | Decreasers  % | Fluctuaters  % | p value |
| --- | --- | --- | --- | --- | --- | --- | --- |
| **Area of residence** |  |  |  |  |  |  | 0.268 |
| Urban | 2694 | 15.1 | 33.0 | 24.3 | 12.9 | 14.7 |  |
| Rural | 3927 | 15.8 | 32.1 | 24.7 | 12.6 | 14.8 |  |
| Remote | 233 | 19.3 | 24.5 | 25.3 | 16.3 | 14.6 |  |
| **Education** |  |  |  |  |  |  | <0.001 |
| No formal education | 743 | 23.2 | 23.3 | 21.8 | 16.2 | 15.6 |  |
| School certificate | 3052 | 16.0 | 30.4 | 24.2 | 13.1 | 16.3 |  |
| Higher school certificate | 2666 | 13.1 | 36.9 | 25.8 | 11.1 | 13.1 |  |
| **Marital status** |  |  |  |  |  |  | 0.041 |
| Married/de facto | 5245 | 15.02 | 32.4 | 25.0 | 12.8 | 14.9 |  |
| Separated/divorced/widowed | 1497 | 16.83 | 31.5 | 23.1 | 13.6 | 15.1 |  |
| Never married | 177 | 24.3 | 29.9 | 22.0 | 12.4 | 11.3 |  |
| **Health care cards** |  |  |  |  |  |  | <0.001 |
| Yes | 3302 | 17.2 | 29.7 | 24.2 | 13.7 | 15.1 |  |
| No | 3626 | 14.2 | 34.4 | 24.8 | 12.2 | 14.5 |  |
| **Body mass index** |  |  |  |  |  |  | <0.001 |
| Underweight / Normal | 2438 | 11.0 | 41.9 | 24.1 | 10.7 | 12.3 |  |
| Overweight | 2292 | 14.9 | 31.3 | 25.6 | 12.8 | 15.4 |  |
| Obese | 1992 | 22.1 | 21.3 | 23.5 | 15.7 | 17.4 |  |
| **Smoking status** |  |  |  |  |  |  | <0.001 |
| Never-smoked | 4374 | 14.8 | 33.2 | 24.9 | 12.2 | 15.0 |  |
| Ex-smoker | 2125 | 15.1 | 32.2 | 24. 6 | 13.8 | 14.3 |  |
| Smoker | 431 | 26.7 | 21.1 | 20.2 | 16.0 | 16.0 |  |
| **Alcohol** |  |  |  |  |  |  | <0.001 |
| Low risk drinker | 3849 | 12.5 | 36.3 | 25.8 | 12.1 | 13.3 |  |
| Non-drinker | 1084 | 20.0 | 26.9 | 20.2 | 15.6 | 17.3 |  |
| Rarely drinks | 1560 | 19.7 | 27.2 | 24.4 | 12.4 | 16.3 |  |
| Risky/high risk drinker | 411 | 18.7 | 26.8 | 24.1 | 15.3 | 15.1 |  |
| **Self-reported health** |  |  |  |  |  |  | <0.001 |
| Excellent/very good | 3342 | 9.6 | 41.3 | 27.0 | 10.0 | 12.18 |  |
| Good | 2707 | 18.0 | 26.6 | 23.8 | 14.1 | 17.5 |  |
| Fair/poor | 891 | 31.3 | 14.7 | 17.1 | 20.3 | 16.6 |  |
| **Number of chronic health conditions** |  |  |  |  |  |  | <0.001 |
| 0 | 1301 | 10.6 | 38.5 | 25.8 | 10.8 | 14.3 |  |
| 1-2 | 3671 | 14.5 | 33.8 | 25.1 | 12.2 | 14.4 |  |
| ≥3 | 1663 | 21.4 | 24.1 | 22.9 | 15.4 | 16.2 |  |

**Supplementary Table 2.** Comparison of **s**ociodemographic, health conditions and physical activity levels in the analytical sample and those lost to follow up. (All data are from the baseline survey in 1996, unless indicated)

|  | **Participants**  (N=6,953) | |  | **Missing or lost to follow up**  (N=6,761) | |
| --- | --- | --- | --- | --- | --- |
|  | N | % |  | N | % |
| **Sociodemographic characteristics** |  |  |  |  |  |
| *Area of residence* |  |  |  |  |  |
| Urban | 2,492 | 35.8 |  | 2,496 | 36.9 |
| Rural | 4,039 | 58.1 |  | 3,759 | 55.6 |
| Remote | 422 | 6.1 |  | 506 | 7.5 |
| *Education* |  |  |  |  |  |
| No formal education | 896 | 12.9 |  | 1,586 | 23.7 |
| School only | 3,316 | 48.1 |  | 3,287 | 49.2 |
| Post School | 2,693 | 39.0 |  | 1,798 | 26.9 |
| *Marital Status* |  |  |  |  |  |
| Married/de facto | 5,900 | 85.2 |  | 5,411 | 80.6 |
| Separated/divorced/widowed | 822 | 11.8 |  | 1,068 | 15.9 |
| Never married | 206 | 3.0 |  | 239 | 3.5 |
| *Health care card ^a^* |  |  |  |  |  |
| Yes | 1,162 | 16.7 |  | 1,064 | 25.2 |
| No | 5,759 | 83.2 |  | 3,153 | 74.8 |
| **Health risks** |  |  |  |  |  |
| *BMI* |  |  |  |  |  |
| Underweight / Normal | 3,673 | 54.3 |  | 3,191 | 50.4 |
| Overweight | 1,899 | 28.1 |  | 1,866 | 29.5 |
| Obese | 1,189 | 17.6 |  | 1,271 | 20.1 |
| *Smoking* |  |  |  |  |  |
| Never-smoked | 3,840 | 56.5 |  | 3,209 | 49.5 |
| Ex-smoker | 1,985 | 29.2 |  | 1,791 | 27.6 |
| Smoker | 965 | 14.3 |  | 1,478 | 22.9 |
| *Alcohol* |  |  |  |  |  |
| Low risk drinker | 5,475 | 79.5 |  | 4,766 | 72.5 |
| Non-drinker | 861 | 12.5 |  | 1,202 | 18.2 |
| Rarely drinks | 204 | 2.9 |  | 243 | 3.7 |
| Risky drinker / High risk drinker | 346 | 5.1 |  | 370 | 5.6 |
| *Perceived health* |  |  |  |  |  |
| Excellent/Very good | 3,728 | 54.0 |  | 2,888 | 43.4 |
| Good | 2,621 | 37.9 |  | 2,757 | 41.4 |
| Fair/Poor | 555 | 8.0 |  | 1,005 | 15.2 |
| *Number of chronic health conditions* |  |  |  |  |  |
| 0 | 2,018 | 30.5 |  | 1,931 | 30.3 |
| 1-2 | 3,566 | 54.0 |  | 3,305 | 52.0 |
| ≥3 | 1,027 | 15.5 |  | 1,119 | 17.6 |
| *Physical activity level ^b^* |  |  |  |  |  |
| Inactive | 2,865 | 44.1 |  | 2,369 | 50.1 |
| Active | 3,630 | 55.9 |  | 2,362 | 49.9 |

^a^ data are from Survey 3 (2001); ^b^ data are from Survey 2 (1998), as these questions were not asked at baseline.
