# Supplementary material for: Twelve year trajectories of physical activity and health costs in mid-age Australian women
Source: Int J Behav Nutr Phys Act. 2020 Aug 10;17:101. doi: 10.1186/s12966-020-01006-6 (PMC7418418; doi:10.1186/s12966-020-01006-6)
Supplement: Supplementary file 2 — Additional file 2: Supplementary Figure 1. Flow diagram showing the selection of participants for inclusion, Australia, 2001-2013 [file 12966_2020_1006_MOESM2_ESM.docx]

**Supplementary Figure 1.** Flow diagram showing the selection of participants for inclusion, Australia, 2001-2013

**
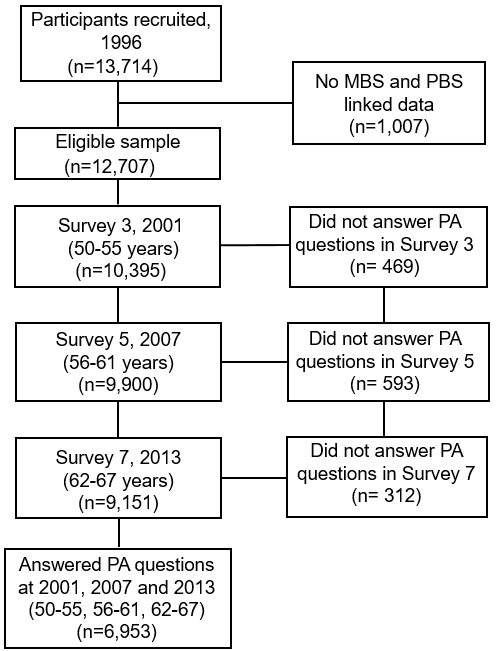
**
